# Marriage and quality of life during COVID-19 pandemic

**DOI:** 10.1371/journal.pone.0256643

**Published:** 2021-09-08

**Authors:** Fredrick Dermawan Purba, Asteria Devy Kumalasari, Langgersari Elsari Novianti, Lenny Kendhawati, Afra Hafny Noer, Retno Hanggarani Ninin

**Affiliations:** 1 Department of Developmental Psychology, Universitas Padjadjaran, Jatinangor, Indonesia; 2 Center for Psychological Innovation and Research, Universitas Padjadjaran, Jatinangor, Indonesia; 3 Department of Clinical Psychology, Universitas Padjadjaran, Jatinangor, Indonesia; 4 Department of Social Psychology, Universitas Padjadjaran, Jatinangor, Indonesia; The University of Hong Kong, HONG KONG

## Abstract

COVID-19 pandemic has impacted people around the globe. Countries, including Indonesia, implemented large-scale social restrictions. Since marriage is found to be beneficial to people’s quality of life (QoL), the study aimed to examine the QoL of married people in Indonesia during a large-scale social restriction of the COVID-19 pandemic. An online cross-sectional survey using Qualtrics was conducted in June 2020. Respondents’ sociodemographic data, spouse data (as reported by the respondents), and pandemic-related data were collected, followed by QoL data, measured by WHQOOL-BREF. WHOQL-BREF consists of 26 questions grouped into four domains: physical, psychological, social relationships, and environmental. Mann-Whitney U, Kruskal-Wallis H and Spearman correlation analyses were employed to compare QoL between groups of sociodemographic characteristics. In total, 603 respondents were recruited. The respondents’ mean age is 35.3 years (SD = 7.61), most are females (82%), bachelor degree graduate (95%), Islam (78%), employed (69%), and assigned to work from home during the pandemic (76%). Married men reported better QoL in almost all domains than women; employed respondents reported higher QoL scores than unemployed; higher educated respondents reported higher QoL than those with lower education; respondents with higher income reported higher QoL than those with lower income. We found significant positive correlations between the QoL scores and age, spouse’s age, and marriage length, although they were considered small. Compared to Indonesian population normative scores pre-pandemic, our sample reported no difference in physical and social domains, lower in the psychological domain, but higher in the environmental domain. Indonesian married people, especially women, those with low level of education, currently out of work, and below-average financial condition are the ones who reported worse quality of life during the lockdown. These results can help direct the Indonesian government efforts in dealing with psychosocial problems during the COVID-19 pandemic, especially for married couples.

## Introduction

COVID-19 pandemic has impacted people around the globe. As of August 31st, 2020, the World Health Organization (WHO) reported 24,854,140 confirmed cases and 838,924 deaths. The worldwide impact of COVID-19 is severe and has become the deadliest pandemic caused by a virus in the last 100 years [1].

To mitigate the spread of the virus, countries around the world have adopted various strategies. Indonesia, notably, adopted large-scale social restrictions (or known as Pembatasan Sosial Berskala Besar [PSBB] in the Indonesian language). This policy measure restricts citizens’ activities in a specific area with a suspected infection and/or contamination to avoid spreading [2]. This so-called ’lockdown’ policy is found to slow the growth of infection [3].

However, the implementation of this policy has some impediments on citizen’s life such as detachment from family and friends, shortages of food and medicine, wage loss, social isolation due to quarantine or other social distancing programs, and school closure [4]. The consequence of these changes in people’s mental health is evident: depression, anxiety, anger, and other stress disorder [5–7]. Beyond mental health, people’s physical health also impacted, including sleep disturbance, physical inactivity, weight gain, insufficient sunlight exposure [7, 8]. The policy also prompted problems in social relations, such as loneliness [9]. However, some people affirmed that their relationships with friends and family members improved, which they could share feelings and caring with their family members and others [10]. Studies reported the positive effect of the pandemic on the environment’s quality, especially in big cities: fewer activities led to improving air quality [11, 12].

The four aspects experienced by individuals during large-scale social restrictions implemented in their respective countries can be explained by the construct of quality of life (QoL): physical health, psychological functioning, social relationship, and environment [13, 14]. The WHO defines QoL as "an individual’s perception of their position in life in the context of the culture and value systems in which they live and concerning their goals, expectations, standards, and concerns" [15]. It is presumed that people’s quality of life is negatively impacted by the COVID-19 pandemic, which is supported by several studies [10, 16].

Earlier studies suggested that those who are married or in a stable long term partnership is healthier and more satisfied with their lives [17]; less likely to undergo anxiety or depression [18] and have better psychological and emotional well-being than those who are single and divorced [19]. In other words, it is confirmed that marriage has a positive impact on the quality of life. However, it is still unclear whether the aforementioned benefits of being married might serve as buffer to the stresses inflicted by the COVID-19 pandemic and the lockdown policy as a measure to the pandemic. Several reports indicated increasing domestic violence [20] during the pandemic with various reasons: economic instability, alcohol, abuse, and weaker women’s support network [21]. Therefore, this primary objective of this study was to examine the QoL of married people in Indonesia during the pandemic, under large-scale social restriction policy. Specifically, we aimed to:

- measure the physical health, psychological functioning, social relationship, and environment domains of QoL among the Indonesian married people,
- spot the sociodemographic characteristics that associated with the quality of life,
- compare the quality of life of Indonesian married people during lockdown policy with QoL of Indonesian general population before the COVID-19 pandemic.

## Materials and methods

### Study design and respondents

We carried out an online cross-sectional survey using the Qualtrics Survey among Indonesian who are married and follow a large-scale social restriction implemented by Indonesia’s local and national government. Inclusion criteria were: (i) married; (ii) aged 18 years, and above; (iii) living in the same residence with the spouse during the COVID-19 pandemic; and (iv) occupying an adequate command of the Indonesian language (Bahasa Indonesia). The survey was conducted from 1 June 2020 to 7 June 2020.

### Procedures

The project was approved by the Universitas Padjadjaran Ethical Committee (No. 514/UN6.KEP/EC/2020). We composed an announcement that provided a general description about the study, of what was expected from prospective respondents, and the study link. We posted this announcement on our social media accounts (e.g., Facebook, Instagram) and shared to our networks using instant messenger applications (e.g., WhatsApp, Line, Facebook Messenger). People who were interested to participate then clicked on a link at the bottom of the announcement and were directed to the online questionnaire. The first part of the online questionnaire is the screening questions, which served to screen out respondents who did not match the inclusion criteria. Those who matched the inclusion criteria then gave their consents and then completed the questionnaires. A reward of IDR 50,000 (approx. 3.4 USD) was transferred to their e-wallet account upon their participation.

### Instruments

We collected socio-demographic data pandemic-related data, including age, gender, length of the marriage, educational level, religion, occupation, financial condition, and work-from-home arrangement.

Quality of life (QoL) was measured by the Bahasa Indonesia version of WHOQOL-BREF provided by the WHOQOL Group [14]. This questionnaire comprises 26 questions, two of which measure the overall quality of life and general health. The other 24 questions were grouped into four domains: physical, psychological, social relationships, and environmental. Each individual item of the WHOQOL-BREF is scored from 1 (very poor/very dissatisfied/not at all) to 5 (very good/very satisfied/extremely) on a response scale. The scores were then transformed into a continuous scale between 0 and 100, with 0 being the least favorable quality of life and 100 being the most favorable [22]. The Indonesian version of the WHOQOL-BREF is available and has been proven to be a valid and reliable questionnaire in Indonesia [23, 24]. For our sample, the internal consistency of the WHOQOL-BREF domains were 0.83, 0.73, 0.68 and 0.78 for physical, psychological, social relationships, and environmental domains, respectively.

### Statistical analyses

Descriptive statistics were used to describe the sociodemographic characteristics of respondents: categorical data (i.e., gender, education level, spouse’s education level, religion, current financial condition, current financial condition compared to before pandemic, have a job, working from home during pandemic) were analyzed using cross-tabulation (frequency and percentages), while continuous data (i.e., age, spouse’s age, length of marriage) were analyzed using means and standard deviations (SD).

QoL data were obtained from the WHOQOL-BREF. We calculated mean scores and standard deviation (SD) of QoL domains in the total sample and subgroups by socio-demographic characteristics. Normality of data was assessed using Shapiro-Wilk test. Since all QoL domain scores were significantly deviate from a normal distribution, we used nonparametric tests. To investigate any association between sociodemographic characteristics and QoL domain’s scores, different tests were calculated. For socio-demographic characteristics with two groups (i.e., gender), Mann-Whitney U (Wilcoxon rank-sum) test was used; and for more than two groups (i.e., education, financial condition), the Kruskal-Wallis H test was used. For sociodemographic characteristics with continuous type of data (i.e., age, spouse’ age, and length of marriage), Spearman correlation analysis was calculated.

Lastly, we compared quality of life domains reported by our sample with the Indonesian population norm results reported by Purba et al from a representative sample of 1046 people aged 17–75 years [24]. This was done to review any significant difference between the respondents’ QoL during the COVID-19 pandemic and the Indonesian population QoL before the pandemic.

We applied the following guideline for strength of the correlation coefficients: <0.3 = small, 0.3–0.5 = medium, >0.5 = large [25], with P-value <0.05 is considered statistically significant. Data management and analyses were conducted using Stata 13 (StatCorp LP, College Station, TX) software.

## Results

In total, 603 respondents completed the online questionnaires and were incorporated in the final dataset (see Table 1). No respondent was excluded. Majority of the respondents are 35.3 years old (SD = 7.61), female (82%), bachelor degree graduate (95%), Islam (78%), employed (69%), and assigned to work from home during the pandemic (76%). The majority of the respondents stated that their financial condition during the pandemic is equivalent to (46%) or higher than other people’s (40%) and claimed that there had been no change of financial condition due to the pandemic (52%).

**Table 1:** Socio-demographic characteristics of respondents.

Characteristics		Mean	SD
Age		35.29	7.61
Spouse’s age		36.64	7.79
Length of marriage		8.69	6.94
		N	%
Gender	Male	108	17.91
	Female	495	82.09
Education level	High school	31	5.14
	Bachelor	363	60.20
	Master/Doctoral	209	34.66
Spouse’s education level	High school	67	11.11
	Bachelor	397	65.84
	Master/Doctoral	139	23.05
Religion	Islam	469	77.78
	Christian	123	20.40
	Others	5	0.82
	Not answer	6	1.00
Current financial condition	Above average	242	40.13
	Average	283	46.93
	Below average	78	12.94
Current financial condition compared to before pandemic	Better	92	15.26
	Similar	316	52.40
	Worse	195	32.34
Have a job	No	184	30.51
	Yes	419	69.49
Working from home during pandemic*	Yes	320	76.37
	No	27	6.45
	In combination	72	17.18

* Only from respondents that have a job (N = 419)

The respondents’ mean scores for each QoL domains, differentiated by subgroups of sociodemographic characteristics, are presented in Table 2. Our respondents reported that their physical functioning domain was the highest and social domain as the lowest. Married men reported better QoL in most domains than women: physical, psychological, and social functioning. Higher educated respondents reported better physical, psychological, and environmental QoL scores than lower educated respondents. Those who are currently employed reported higher QoL scores than those who were unemployed. Concerning current financial condition, those who considered themselves as above average reported the highest scores in all domains of QoL compared to those who considered their financial condition as below average. Concerning work arrangement, we found no significant differences of QoL scores of any domains between work-from-home, work-from-office or combination arrangement during the pandemic.

**Table 2:** Mean scores and standard deviation (SD) of quality of life domains in the total population sample and sub-samples by socio-demographic characteristics.

Characteristics	Physical		Psychological		Social		Environment	
	Mean	SD	Mean	SD	Mean	SD	Mean	SD
All	67.82	11.93	64.32	13.18	63.29	14.73	64.13	12.36
Gender								
Male	70.04*	11.86	68.60*	10.99	66.59*	15.85	63.72	12.54
Female	67.33*	11.90	63.39*	13.44	62.58*	14.39	64.22	12.33
Education level								
High school	65.32*	13.31	60.08*	15.13	60.22	17.57	56.35*	15.53
Bachelor	67.23*	11.80	63.90*	13.22	63.25	14.89	62.69*	11.84
Master/Doctoral	69.21*	11.87	65.69*	12.66	63.84	14.00	67.78*	11.74
Spouse’s education level								
High school	66.95	12.69	63.25	13.61	62.56	16.44	57.84*	12.79
Bachelor	67.24	11.16	64.29	13.02	63.22	14.21	63.75*	11.60
Master/Doctoral	69.89	13.45	64.93	13.46	63.85	15.40	68.23*	12.88
Current financial condition								
Above average	70.34*	11.97	66.89*	12.17	65.32*	14.96	70.42*	10.64
Average	66.41*	10.99	63.27*	12.65	62.19*	13.86	61.31*	10.29
Below average	65.11*	13.69	60.20*	16.29	61.00*	16.42	54.85*	14.58
Current financial condition compared to before pandemic								
Better	67.04	12.34	65.44	13.89	62.59	14.80	64.06*	10.83
Similar	68.34	12.51	64.78	13.56	63.74	15.19	66.46*	12.17
Worse	67.33	10.74	63.06	12.14	62.91	13.98	60.38*	12.49
Have a job								
No	65.49*	11.90	61.55*	14.36	60.82*	14.79	62.01*	12.65
Yes	68.84*	11.81	65.54*	12.45	64.38*	14.59	65.06*	12.13
Working from home during pandemic*								
Yes	68.84	11.56	65.72	12.44	64.45	14.79	65.63	11.94
No	69.71	13.85	66.36	12.71	65.74	12.30	62.85	12.69
In combination	68.50	12.29	64.47	12.49	63.54	14.63	63.37	12.69

* Using Mann-Whitney U (Wilcoxon rank-sum) test for two groups or Kruskal-Wallis H test for more than two groups, the mean score between the demographic subgroups differs statistically significant, p-value <0.05

Table 3 shows that respondents’ age is correlated positively with all domain scores. The spouse’s age and marriage length are positively correlated with three domain scores: physical, psychological, and environment. However, these correlations are considered small (rho<0.3).

**Table 3:** Spearman correlation test between quality of life domains and age and length of the marriage.

Characteristics	Physical		Psychological		Social		Environment	
	Rho	P-value	Rho	P-value	Rho	P-value	Rho	P-value
Age	0.198	<0.001	0.168	<0.001	0.082	0.045	0.163	<0.001
Spouse’s age	0.187	<0.001	0.137	0.001	0.071	0.083	0.144	<0.001
Length of marriage	0.182	<0.001	0.160	<0.001	0.063	0.120	0.163	<0.001

We compared our sample’s QoL during pandemic time with Indonesian general population normative scores pre-pandemic. We found no significant differences in physical and social domains. Our sample reported lower psychological domain scores but higher environmental domain scores than the Indonesian general population (see Table 4).

**Table 4:** Comparison between quality of life domains and population norm scores.

Domains	Mean	SD	Population norm*	P-value
Physical	67.82	11.93	69.23	0.1452
Psychological	64.32	13.18	66.74	<0.0001
Social	63.29	14.73	63.13	0.0710
Environment	64.13	12.36	58.53	<0.0001

* as presented in Purba et al. from 1046 respondents age 17–75 years [24]

## Discussions

This study aimed to investigate Indonesian populations’ quality of life during the COVID-19 pandemic, especially those married and living in the same household with their spouse. It was discovered that the physical domain has the highest score among the four QoL domains, while the social domain was the lowest. Sub-groups of gender, education level, having a job, and current financial condition reported significant differences in the domain scores: e.g., those who are employed perceived their financial condition during a pandemic to be better than others.

Our study found that quality of life during the implementation of a large-scale social restriction depends on gender, education level, job status, and financial condition. This finding is similar to the results in the general Indonesian population [24] and the general population of Denmark, Southern Brazil, and Australia, in which all components were measured using the WHOQOL-BREF, before the COVID-19 pandemic [26–28].

Men reported higher QoL in almost all domains than women. This finding is similar to reports from previous studies that measured quality of life in general population [24, 29, 30]. In Indonesia pre-pandemic, more women face mobility problems, daily activity dilemmas, and pain or discomfort [24]. Another study reported that almost two-third of the parents who experienced high-stress levels in the pandemic time are women [31]. The condition is similar to finding in Italy that women, compared to men, are associated with higher depression levels during lockdown [32]. Hobbins et al. argued that this might be related to different exposure to health risks (such as more stressful jobs or working environment, work-family conflict) and greater vulnerability to health risks (such as lower access to health resources) [33]. Amid pandemic, the lower QoL of women might be related to a woman’s roles in a household: a wife, a mother, and the one responsible for domestic responsibilities; women spend more time than men on domestic work (UN Women, 2020). When everyone stayed at home because of social restriction, women’s role becomes more burdensome. Indonesian mothers are expected to bear the responsibility of child-rearing and children’s school activities, carrying out domestic chores, and taking care of all family members [34].

Respondents with higher education level reported better QoL in almost all domains, except for the social domain. According to studies measuring life quality [24, 35], this condition is common, although those studies were conducted before the pandemic. In contrast, a study in Italy asserted contradictory results: higher education respondents stated higher anxiety than lower education, but that was not the case for depression and sleep disturbance [7]. It could be argued that respondents with a higher level of education read more about the virus and pandemic that led to anxiety. However, they better understand the COVID-19 pandemic and various national and personal mitigation efforts, therefore hindering them from further problems such as depression and sleep disturbance.

Being employed led to better QoL. Similar findings were also recorded in other studies [27, 36, 37]. Job insecurity is found to have immediate detrimental effects of job insecurity on the physical (e.g., somatic symptoms, pain), psychological (e.g., anxiety, depression, affect), and social functioning (e.g., social support, marital discord) of employees [38]. A study in the United Kingdom found that having a job is a significant protective factor for general psychiatric disorders and loneliness [9]. One compelling finding concerning financial condition is that about half of the respondents reported that their current financial condition is similar to prior the pandemic. Likewise, such a situation was found from a study in China where about three-fourth of their respondents mentioned that they did not face financial stress arising from the pandemic [10].

We also found significant correlation between the respondents’ and their spouses’ age and their length or marriage with almost all QoL domain scores, even though the correlations are considered small. This is in line with a study by Conversano et al that found that older-age individuals showed less psychological distress during the COVID-19 pandemic. They also found that living with one’s spouse predicted lower distress during the COVID-19 quarantine because the respondents were feeling protected in terms of psychological wellbeing [39].

Although the sample in the present study is different from the Indonesian general population sample in a preceding study [24], an indirect comparison of QoL before and during COVID-19 can be conducted. No significant differences were found for physical and social domains. It could be argued that those who were healthy and not contracted by the virus were the ones who participated in this study; therefore, the non-difference in the physical domain is somewhat expected. For the social domain, this further supports the availability and support of spouse, family, and other significant persons during lockdown and pandemic [9, 40]. We also found that our respondents reported lower psychological functioning than the general population before the COVID-19 pandemic. Previous studies also reported a high level of mental health problems such as stress, anxiety, or depression, in different groups because of the pandemic, e.g., college students in China [41], the general population in the United States [40], Italy [7, 42], and the United Kingdom [9]. On the other hand, the respondents reported higher environmental domain scores. This exception might be associated with fewer commutes. Low numbers of vehicles on the streets led to less pollution, hence better air quality, remarkably notable in big cities [11, 12]. Furthermore, they may enjoy the benefit as they have more stable financial security [9, 10].

Taken together, our results could provide an important reflection on the direction of the efforts of the government in their policy development during the COVID-19 large-scale social restriction: aiding married couples (especially the women) who have low level of education, currently out of work, and in below-average financial condition so that they will be able to improve their quality of life.

Several limitations of the present study have to be considered. First, we cannot infer the influence of being married and quality of life during the COVID-19 large-scale social restriction because we have no unmarried respondents as control group. Second, our study is not representative of the overall population of Indonesian married people because the design did not take into account all sociodemographic strata in Indonesia. The nature of an online survey which mainly targeting people who are literate and have access to the internet induces a selection bias in the achievement of the study goals. In the present study, the bias is shown by the high percentage of respondents with higher level of education (at least bachelor graduate) and young adults (mean age = 35 years) of our respondents. Thus, the generalization of the findings to other sociodemographic characteristics, such as lower-level education and older populations should be made cautiously. Third, we did not have the respondents’ pre-pandemic data, therefore no direct comparison of quality of life before and during pandemic can be made in the present study. We compared our respondents’ data indirectly with the Indonesian quality of life normative data, reported by Purba et al [24].

## Conclusions

Large-scale social restriction (lockdown) is one strategy implemented by the governments across the globe, including Indonesia, to mitigate the spread of the coronavirus disease 2019 (COVID-19). Indonesian married people, especially women, those with low level of education, currently out of work, and in below-average financial condition are the ones who reported worse quality of life during the lockdown. These results can help direct the Indonesian government efforts in dealing with psychosocial problems during the COVID-19 pandemic, especially for married couples.
